# Dendritic Cells and Their Role in Allergy: Uptake, Proteolytic Processing and Presentation of Allergens

**DOI:** 10.3390/ijms18071491

**Published:** 2017-07-11

**Authors:** Piotr Humeniuk, Pawel Dubiela, Karin Hoffmann-Sommergruber

**Affiliations:** Department of Pathophysiology and Allergy Research, Center for Pathophysiology, Infectiology and Immunology, Medical University of Vienna, Vienna 1090, Austria; piotr.humeniuk@meduniwien.ac.at (P.H.); pawel.dubiela@meduniwien.ac.at (P.D.)

**Keywords:** allergens, dendritic cells, presentation, processing, uptake

## Abstract

Dendritic cells (DCs) are the most important antigen presenting cells to activate naïve T cells, which results in the case of Type 1 allergies in a Type 2 helper T cell (Th2)-driven specific immune response towards allergens. So far, a number of different subsets of specialized DCs in different organs have been identified. In the recent past methods to study the interaction of DCs with allergenic proteins, their different uptake and processing mechanisms followed by the presentation to T cells were developed. The following review aims to summarize the most important characteristics of DC subsets in the context of allergic diseases, and highlights the recent findings. These detailed studies can contribute to a better understanding of the pathomechanisms of allergic diseases and contribute to the identification of key factors to be addressed for therapeutic interventions.

## 1. Introduction

Dendritic cells (DCs) are the most important antigen presenting cells to activate naïve T cells, and they are therefore the central players of the immune system crossing the bridge between innate and adaptive immunity. To play such an important role and keep the balance between health and disease, they must have a unique set of features that enables them to operate at the interface of host defense and tolerance. Within this review, the most important characteristics of DC subsets in the context of allergic diseases will be described.

Type 1 allergic diseases are called the epidemics of the 21st century, and up to 25% of the population may be affected by allergic symptoms. The symptoms range from local reactions of the mucosa to generalized symptoms in the skin, gastrointestinal tract, airways, and heart and circulation system [1].

The adverse reaction to otherwise innocuous substances is caused by an exaggerated immune reaction. Although this disease was described by Clemens v. Pirquet more than one hundred years ago, the underlying mechanisms have been gradually identified. Allergens are usually (glyco-) proteins with a molecular mass ranging from 5 to 80 kDa. Although it is evident that the allergens identified so far are restricted to a comparatively small number of protein families, there is no common structural motif or protein function that is shared by all known allergens so far [2].

It seems that the genetic background of an individual plays an important role in the development of allergic symptoms. However, the knowledge of certain genes related to allergies is still incomplete. Upon first contact with an individual’s mucosal site with allergens, the sensitization phase is built up. Via the uptake and presentation of allergens by dendritic cells residing in the respective mucosal tissue, naïve CD4^+^ T cells are activated and differentiate into Type 2 helper T cell (Th2) cells. Consecutively, Th2 cells and their cytokine production driven by IL-4 and IL-13 promote/facilitate the production of allergen-specific immunoglobulin E (IgE)antibodies from B cells. During the following elicitation phase the frequent exposure to the same allergen induces mast cell activation via cross-linking of allergen-specific IgE antibodies on the surface and the release of histamine. This in turn causes a variety of symptoms, and can affect different organs. Most frequently, skin reactions are observed, such as urticaria, oedema, and eczema. Rhinoconjunctivitis is commonly seen upon exposure by inhalant allergen sources, and even the lungs can be affected by asthma attacks. Upon ingestion of food allergens, gastrointestinal symptoms such as diarrhea, stomach pain, nausea, and vomiting can occur. The most severe life-threatening reaction is anaphylaxis, characterized by cardiovascular symptoms and involvement of the respiratory tract that requires emergency treatment.

A number of animal models have been developed to study the pathomechanism of allergic diseases and to obtain a better insight into the cellular interplay and orchestration of cytokine production as summarized in excellent reviews [3,4,5]. A range of different allergy animal models have been established including mice, rats, guinea pigs, dogs, pigs, and monkeys. As readout, the immediate response in the animal can be assessed in vivo as well as in vitro after sacrifice. Murine models are most frequently used to study the development of allergic sensitization, elicitation, and the potential of immunotherapeutic interventions. However, the genetic background of different mouse strains has an effect on the development of allergic symptoms. BALB/c mice develop high IgE levels upon allergen sensitization treatment. In contrast, C57BL/6 mice are known to respond by intermediate or low IgE levels. However, the results obtained in an animal model need to be interpreted with caution, and may not be comparable to immune responses in humans [3].

This review summarizes the role of DCs in the context of allergic disease, and the current methods applied to study the uptake, processing, and presentation of allergenic proteins by DCs.

## 2. Dendritic Cells Are the Most Important Antigen Presenting Cells in Health and Disease

Dendritic cells (DCs) were discovered by Ralph Steinman in 1973, and since then, extensive studies have been performed to characterize the role of different subsets, as extensively summarized in excellent reviews [6,7,8].

Classical (also called conventional) DCs (cDCs) represent the prototype of antigen-presenting cells. They are characterized by a number of unique features that enables their eminent function in antigen sampling and presentation, which is facilitated by high expression levels of major histocompatibility complex class II (MHC class II) molecules. Once activated, DCs take up antigens and migrate to the draining lymph nodes in order to prime naive T cells [9]. However, clearly distinguishing such “genuine” cDCs from other cells requires widespread access to state-of-the-art technology such as polychromatic flow cytometry, mass spectrometry, and transcriptional profiling. Following the definition, DCs constitutively express hematopoietic surface makers such as CD45, MHC-II, and CD11c, and lack lymphocyte, granulocyte, and erythrocyte lineage markers. Separation of cDCs and macrophages must be supported by additional markers—i.e., the tyrosine kinase receptor form, tyrosine kinase 3 (lt3, named also Flk2) or CD135) [10]. The role of cDCs as sentinels requires them to constantly sense and respond to environmental stimuli. Due to the specific role, lymphoid and non-lymphoid tissues require functionally distinct DC subsets. In non-lymphoid tissues, 1–5% of the cell population is represented by cDCs (depending on the organ), consisting of two major subsets: CD103^+^CD11b^−^ and CD11b^+^. CD103^+^ cDCs are enriched in the Peyer’s patches of the intestine, also co-expressing CD8. In the lamina propria and muscle, CD11b^+^ cDCs arise from cDC-precursors and monocytes [11,12]. With regard to lymphoid tissue-resident cDCs undergo a differentiation process and spend their entire lives within lymphoid tissues represented by two subsets: CD8^+^ and CD11b^+^ cDCs. In addition, lymph nodes also contain non-lymphoid tissue-migratory cDCs, whereas lymphoid tissue-resident cDCs make up the entirety of the splenic cDC compartment [13].

**Figure 1:**
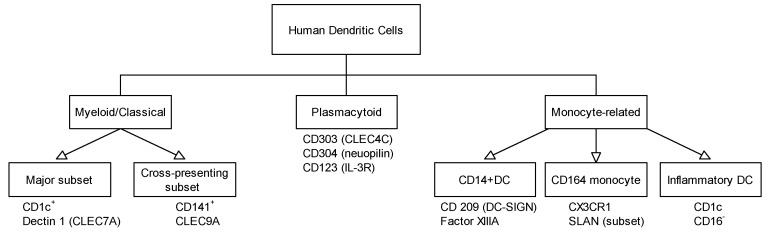
Overview on the major human dendritic cell (DC) populations and their surface markers.

Plasmacytoid dendritic cells (pDCs) represent a small but interesting subset of DCs sharing a common origin with cDCs, but differing in their life cycle. Historically, they were called interferon-producing cells [14]. A summary on surface markers relevant for different DC subsets is presented in Figure 1.

Originally, monocytes were identified as the most relevant players of the mononuclear phagocytic system. They further differentiate into different subgroups of tissue macrophages with additional specific functions [15]. More than two decades ago it was demonstrated that monocytes are able to differentiate into DCs in vivo during inflammation [16]. Therefore, monocyte-derived dendritic cells (moDCs) are key players in both innate and adaptive immunity, due to their anti-bacterial potential combined with their capacity to stimulate CD4^+^ and CD8^+^ T cell responses. Via the CD4^+^ T cell activation, antibody production by B cells is triggered as reviewed by Leon et al., 2008 [17].

Dendritic cells consist of heterogeneous groups, and depending on the location and their origin they can be identified by certain surface markers. A selection of different surface markers specific for cDCs, pDCs, and Langerhans cells (LCs) are summarized in Table 1. While some markers are common for both, others are only specific for either human or murine cells.

## 3. Different Dendritic Cell Subsets Identified in Skin, Respiratory, and Gastrointestinal Tract and Their Role in Allergy

Allergic diseases can affect a number of organs, yet skin, respiratory, and gastrointestinal tract are most frequently involved, since they represent the internal and external barrier to environmental antigens. Dendritic cells are abundant and distributed across the human body, and their localization is strictly reflected by their functions as antigen presenting cells. Immature DCs reside where antigen entrance is expected; that is, the epithelia of the skin and mucosa of the gut, upper and lower airways, and the urogenital system. As for other antigens, allergens must cross the cellular barriers to come into first contact with DCs.

Specialized DCs reside in the skin to quickly sense and sample the antigens to further decide whether the antigen poses a harmful risk. In the skin, the special type of dendritic cell is called Langerhans cells. The demonstration of MHC Class II, Fc, and C3 receptors on epidermal Langerhans cells (LCs) confirmed their identity as DCs, and since their discovery they have been extensively studied [19,20]. However, additional DC subpopulations have also been identified in the mammalian dermis [7].

Immediately after allergens have crossed the skin barrier, epidermal Langerhans cells sample antigens and become activated as shown by the expression of various co-stimulatory molecules, such as CD54, CD80, and CD86. In parallel, enhanced production of proinflammatory cytokines such as IL-1α, IL-12, and TNF-α is triggered [21]. This inflammation process is accompanied by the infiltration and accumulation of moDC in tissues, as shown by animal models. These cells express the murine DC markers (CD11c, CD80, CD86, MHC II, and DEC205) together with monocyte (CD11b, Ly6C) and macrophage-associated markers (Mac-3, F4/80) [22].

In atopic dermatitis, CD1a^+^CD11b^+^CD1c^+^ myeloid DCs and plasmacytoid DCs were observed in the skin [23]. Both subsets express the high affinity IgE receptor, FcεR1 [24,25,26]. In psoriasis, pDCs were identified as an important population of inflammatory lesions [27] attracted by chemerin production provided by dermal fibroblasts, endothelial cells, and mast cells [28]. LCs are also responsible for tolerance induction as shown in the murine model with 2,4-dinitrothiocyanobenzene (DNTB)-treated LCs. After in vivo administration of DNTB-treated LCs to naive recipients, tolerization of the antigen was observed via regulatory T cell (Treg) activation [29].

In human lungs, two major DC populations can be found: myeloid dendritic cells (mDCs) and plasmacytoid dendritic cells (pDCs), respectively. Upon allergen challenges, DCs are recruited to the airway tissues. Immunofluorescent staining of bronchial mucosa sections revealed a significant increase in CD1c1HLA-DR1 DC numbers induced by allergen challenge [30]. In a similar study, increased numbers of mDCs and pDCs were observed in bronchoalveolar lavage fluid (BALF) samples after challenges with dust mite, rye grass, and birch pollen allergens in patients with allergic asthma [31].

Throughout the epithelia of the respiratory tract, including the nose, nasopharynx, large conducting airways, bronchi, bronchioles, and alveolar interstitium, DCs form a solid network for antigen sampling [32,33,34,35,36]. In confluent airway epithelial cells, the enzymatic activity of Der p 1—one of the major house dust mite (HDM) allergens—disrupted the tight junctions and thus destroyed the intact epithelial barrier function [37]. HDM allergens also interact with the receptor dectin-2 on bone marrow-derived murine DCs, resulting in the synthesis and release of cysteinyl leukotrienes by DCs [38]. Via this autocrine mode of action, the release of cysteinyl leukotrienes fuels the allergic inflammation and drives the Th2 polarization continuously. In another murine model of cockroach allergy, the proteolytic activity of cockroach allergens activated PAR-2 on pulmonary mDCs and facilitated Th2/Th17 cytokine production and allergic airway responses [39].

Usually gut-associated DCs play a key role in the induction of Tregs and are therefore responsible for surveillance and tolerance towards harmless and beneficial dietary antigens. However, during an allergic response, DCs sample the allergen either directly from the gut lumen or via specialized microfold (M) cells, and subsequently present the antigen to naive T cells in the Peyer patches or the gut-draining mesenteric lymph nodes (MLNs) [40,41]. Resident DCs in the intestinal mucosa are characterized by the expression of CD11c and CD103. The antigen presentation mechanisms involved in the differentiation of Th2 cells has presumably evolved in response to extracellular multicellular parasites such as helminths, but Th2 cells also play a central role in the pathophysiology of allergic inflammatory diseases [42,43].

Consequently, upon activation, dendritic cells start to lose adhesiveness to their surrounding epithelia and express chemokine receptors to migrate to the T cell dominating zone in the local draining lymph nodes [44]. Depending on the signals they provide to naive T cells, they are polarized into either Th1 or Th2 effector cells and in parallel induce a subset of long-living memory Th cells. Once DCs mature, they reveal very limited processing capacity and are characterized by the expression of high levels of MHC class II molecules and costimulatory molecules (i.e., CD40, OX40L, ICOS, CD80, or CCR7) that provides the second signal for T cell activation [45]. They also secrete a variety of cytokines, such as IL-10, Il-12, and IL-23, that attract different cell types of the immune response [46].

## 4. Antigen/Allergen Uptake by Dendritic Cells 

**Figure 2:**
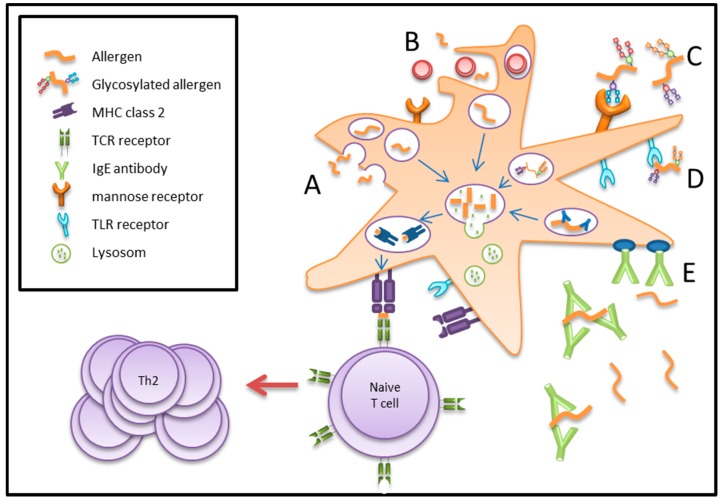
Antigen uptake by DCs via three independent mechanisms of A: Macropinocytosis; B: Phagocytosis; C–E: receptor-mediated endocytosis (mannose receptor C, TLR receptor D, Fcε receptor E). MHC class 2: major histocompatibility complex class II.

Antigen uptake by DCs is executed by three independent mechanisms: macropinocytosis, phagocytosis, and receptor-mediated endocytosis, as outlined in Figure 2. The uptake mechanism determines not only the intracellular trafficking of the antigen, but also the type of T-cell epitopes being generated.

Macropinocytosis is based on a non-specific uptake of antigens, nutrients, and soluble molecules. This seems to be a dominant mechanism also involved in the uptake of pollen allergens by LCs [47]. Immature DCs then recognize antigens by membrane-expressed low-specificity receptors such as Toll-like receptors, and interaction of these receptors with their ligand and inflammatory cytokines produced by innate immune response in parallel elicit a complex signaling cascade. This endocytosis is an actin-dependent formation of macropinosomes. The endocytosed antigens are then transferred into early and late endosomes, which fuse with the antigen processing compartments to ensure MHC-II antigen presentation. Macropinocytosis is a constant sampling process by DCs and is not restricted to an active immune response. In fact, it is the checkpoint for whether an immune response needs to be switched on or peripheral tolerance induction is continued in a healthy status. Using ovalbumin (OVA) peptides and exposing them to peripheral plasmacytoid DCs in the thymus led to clonal depletion of OVA-specific thymocytes, thus also contributing to central tolerance [48,49].

In contrast, phagocytosis internalizes large antigens, apoptotic cells, and opsonized pathogens. The process is initiated by the activation of surface molecules. However, non-specific phagocytosis can also be performed. Antigens are endocytosed by membrane-derived phagosomes and further processed in phagolysosomes.

In receptor-mediated endocytosis, specific receptors on the cell surface such as C-type lectin carbohydrate receptors (i.e., langerin, DC-SIGN, BDCA-2, mannose receptor) [50,51,52], Fcγ (i.e., CD32, CD64), and Fcε receptors [53] are involved. Subsequently, these molecules are transported by clathrin-coated vesicles or non-clathrin-coated vesicles. Upon uncoating of clathrin, antigens are delivered to early endosomes [54]. Polymorphic MHC-II complexes are formed in antigen processing compartments and subsequently guided to the plasma membrane in order to initiate the antigen-specific immune response via T-cell interaction. Interestingly, lectin receptor-mediated uptake by DCs results in a highly efficient presentation to T cells (up to 100-fold increase) as compared to antigen internalization via fluid phase [55,56].

In the context of allergic diseases, it is largely unknown what the major differences between healthy and atopic individuals are when being exposed to allergens and sampling by DCs starts. Allergen uptake and processing by DCs is a pivotal step in the induction of Th2 responses. It is known that human DCs are involved in the Th2 sensitization process against common allergens in individuals with genetic predisposition [57]. The Western life style has been described as a reduced exposure of environmental microbial load to the human immune system. As a consequence, this absence of microbial stimulation could explain why DCs are not instructed to produce IL-12 and fail to counteract Th2 responses. However, other studies suggest that there is no difference in IL-12 production in moDCs between atopic and non-atopic individuals [58]. A third theory investigated whether allergen exposure levels contribute to an allergic immune response. Chronic low-level exposure to inhaled allergens can lead to decreased levels of DCs continuously migrating to the lymph nodes and inducing sustained TCR ligation at low stimulator/responder ratios, conditions known to induce Th2 responses [59,60]. The first encounter of DCs with allergens can be investigated by using monocyte-derived DCs (moDCs) obtained from healthy and allergic individuals, respectively. Peripheral blood monocytes are cultured for 7 days with IL-4 and granulocyte macrophage-colony stimulating factor (GM-CSF) and then exposed to fluorescent-labelled allergens. In vitro, moDCs have been shown to upregulate surface markers (i.e., CD80, CD83, CD86, and HLA-DR) after stimulation with lipopolysaccharides [61,62] and major allergens from birch pollen (Bet v 1, [63,64,65]), peach (Pru p 3, [66]), peanut (Ara h 1, [67]), house dust mite (Der p 1, [68]), and grass pollen (Phl p 5, [69]). When moDCs were incubated with purified HDM and birch pollen allergens (Der p 1 and 2, Bet v 1) and subsequently cultured with autologous T cells, they induced predominantly Th2 responses only in the allergic donors, despite similar IL-12 expression levels in both study groups [70,71]. Other studies showed that high affinity IgE receptor FceRI was upregulated in CD1a^+^ Langerhans cells in patients with atopic dermatitis and in DCs in the nose and bronchi of allergic patients [24]. However, recent studies showed that IgE-facilitated uptake is not necessary for Bet v 1 internalization of moDCs [69,72] and blocking endocytic pathways inhibits Bet v 1 uptake [72]. Interesting results regarding allergen uptake come from Al-Ghouleh et al. They showed that the uptake of the glycosylated allergen Der p 1 is carbohydrate-dependent and was performed via the mannose receptor. Reduction in the uptake due to allergen deglycosylation indicates that glycan moieties play a crucial role in their recognition by innate immune cells, contributing to a downstream activation of Th2 cells and IgE production [73].

## 5. Antigen/Allergen Processing and Presentation

**Figure 3:**
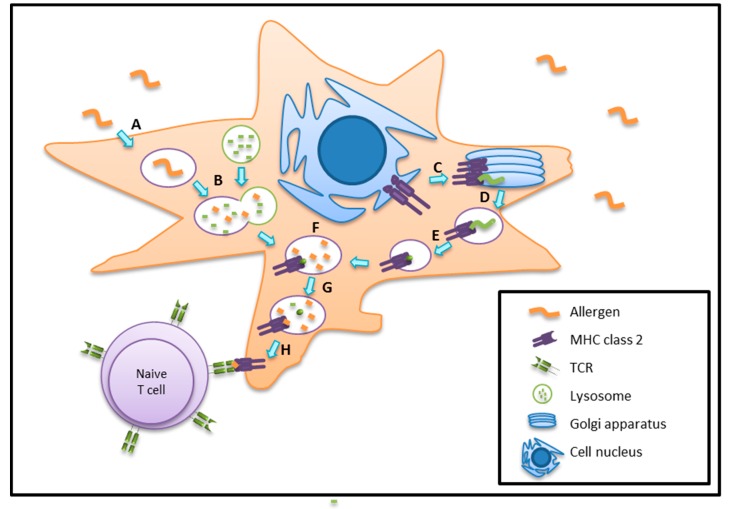
Processing of allergens requires a stepwise degradation followed by loading of MHC II molecules and allergen presentation to T-cell receptors (TCRs) A: Allergen uptake; B: Phagolysosome generation; C: Transfer of MHC class II from endoplasmic reticulum (ER) to Golgi apparatus; D: Forming vesicle with MHC class II; E: Degradation of invariant chain (li); F: Fusion of phagolysosome with MHC class II containing vesicle; G: Release of class I-associated invariant chain peptide (CLIP) and peptide binding; H: Allergen presentation.

Subsequent processing and lysosomal degradation of allergens is crucial, and the most resistant peptides are then being presented via MHC II molecules to the T cell receptor ((TCR); Figure 3). The antigens sampled by DCs are finally processed in late endosomal or lysosomal structures that are enriched in proteolytic enzymes and disulphide reductases [74]. These proteases are activated by a rather low pH environment. This degradation process is tightly regulated, and includes asparaginyl endopeptidase and cathepsin S. Cathepsin S, B, H, and L are also important for promoting peptide MHC II complex assembly. The class I-associated invariant chain peptide (CLIP) remains bound to the MHC II peptide binding groove. Later on, CLIP is released to facilitate the binding of antigenic peptides to the MHC II, and finally the antigenic peptide is guided to the surface to be presented to naïve CD4^+^ T cells [74].

Interestingly, the lysosomal extracts differ in their activity; i.e., enzymatic cocktails from DCs are 50 times less active compared to those from macrophages. Therefore, DCs can preserve internalized antigens in intact form for some hours in vivo [74]. Another mechanism regulates the balance between antigen proteolysis and the complete destruction of antigenic structures in DCs, via NADPH oxidase 2 (NOX2). Involvement of NOX2 increases phagosomal pH, which in turn prolongs antigen presentation by MHC II.

Based on recent findings, it seems that individual food allergens—even if they share structural features—display different resistance to DC processing. As shown by Schulten et al., two allergens from the non-specific lipid transfer protein (nsLTP) family displayed different stability when subjected to lysosomal degradation assays. Cor a 8, from hazelnut, was more rapidly degraded as compared to Pru p 3, the major allergen from peach. A similar observation was found for the birch pollen (Bet v 1) homologues from food sources. Dau c 1 from carrot showed remarkable stability as compared to Api g 1 from celery [75].

Interestingly, DCs can also process glycolipid antigens, as for example disaccharide Gal(α1→2)GalCer. To stimulate T-cell receptors (TCRs), the terminal sugars have to be removed. A lysosomal enzyme—α-galactosidase A—is required for the generation of the antigenic monosaccharide epitope [76].

Allergen presentation is another crucial task performed by DCs. This process takes place when DCs have reached the T cell zone of the draining lymphoid organ. However, circulating DCs may also engage other DCs to present the same antigen in the lymph nodes. The transfer occurs either by phagocytosis of the antigen loaded DC [77] or by the release of antigen-bearing vesicles (exosomes) [78]. After migration, DCs stop allergen sampling and start producing a range of chemokines (MIP3-b, chemokine C-C motif ligand 18 (CCL18) monocyte-derived chemokine, thymus and activation regulated chemokine) to attract naive and resting T cells [79]. DCs can present allergenic proteins bound to the major histocompatibility complex class II (MHC class II) to the T-cell receptor (TCR). In immature cells, new MHC class II molecules accumulate in late endosomes and lysosomes, while in mature DCs, class II molecules are present at the cell surface [80,81]. Upon recognition of the TCR and the interaction of co-stimulatory molecules present on DCs and T cells, signaling activates the respective T cell. One of the best characterized co-stimulatory molecules expressed by DCs are CD80 (B7-1) and CD86 (B7-2). Both of them interact with CD28 expressed on the surface of T cells [77,80].

In the recent past, lipids and their potential role during the allergic sensitization process were also investigated. DCs using CD1 proteins are able to present lipid antigens to specific T cells. CD1 surface molecules represent a family of transmembrane glycoproteins expressed in association with β2-microglobulin on the cell surface of antigen presenting cells (APCs) together with bound lipid or glycolipid ligands [82]. Two groups of CD1 molecules exist: group 1 consists of CD1a, b, and c molecules, while the CD1d molecule belongs to group 2. CD1a, b, and c are part of the defense against microbes [83]. The CD1d molecule activates NKT cells (Natural killer T lymphocytes) to produce a huge range of cytokines, such as INF-γ, IL-4, IL-13, and IL-17 [84]. For some allergenic proteins, it has been shown that the food matrix—especially rich in lipids—can affect the immune responses. For example, the food matrix of peanuts was crucial for the immunostimulatory activity of purified peanut allergens. Only the complete peanut extract induced an increase in cell number, cytokine production, and activation of antigen-presenting cells [85]. Additionally, in a murine allergy model, for major brazil nut allergen (Ber e 1), a specific lipid fraction was required to induce an allergic immune response [86]. Finally, Abos-Gracia investigated the effect of lipids obtained from olive pollen on DCs. Upon interaction of the lipid fraction with moDCs, upregulation of surface molecules CD1d and CD86 was observed [87].

## 6. Conclusions

As professional antigen presenters, dendritic cells are of crucial importance for antigen recognition, uptake, and presentation to naïve T cells for stimulation or priming. During an allergic response, sampling of allergens leads to a Th2 polarization with subsequent cytokine expression patterns. Therefore, methods to study the uptake, processing, and presentation of individual allergens have been developed to identify the relevant key factors that discriminate between an active Th2-driven response versus a tolerogenic status. Recent findings suggest that in addition to protein conformation, additional features such as their protease activity, ability to bind lipids, and activation of toll-like receptors contribute to the allergenic potential of certain proteins. Furthermore, the role of matrix components of allergen sources such as lipids and carbohydrates seem to play a role, via the activation of innate immune responses. Therefore, these detailed studies will provide a better insight into the pathomechanism of Th2-driven immune responses and can elucidate the key points to be addressed for therapeutic actions.

## Figures and Tables

**Table 1:** Comparative functional analysis of murine and human DCs and their specific surface markers; modified from Reynolds et al., 2015 [18]. cDC: classic (or conventional) DC; IFN: interferon; LC: Langerhans cell; pDC: plasmacytoid DC; Th1, Th2, Th17: Type 1, 2 and 17 helper T cells; TLR: Toll -like receptor; Tregs: regulatory T cells.

DC Subtype	Human	Cellular Function	Mouse
cDC1	CD141	Cross-presentation; IL-12 and IFN-λ production; Expression of TLR3; Induce Th1/Th2 responses	CD103/CD8
	XCR1		XCR1
	CLEC9A		Clec9A
	CADM1		CADM1
cDC2	CD1c	CD4^+^ T cell responses; IL-1B, IL-6, and IL-23 production; Expression of all TLRs apart from TLR3 (mouse) and TLR9 (human); Induce Th2/Th17 responses	CD24
	CD11b		CD11b
	SIRPα		SIRPα
pDC	CD123	Anti-viral responses; IFN-α production; Expression of TLR7 and TLR9	SiglecH
	CD303		Bst2
	CD304		Ly6c
LC	Langerin CD1a^+++^ CD11c^lo^	Maintain epidermal integrity; Induce Tregs; Induce Th17 responses	Langerin CD24 CD11b F4/80

## Abbreviations

CCL18	Chemokine C-C Motif Ligand 18
CD	Cluster of Differentiation
cDC	Classic Dendritic Cell
CLIP	Class I-Associated Invariant Chain Peptide
DC	Dendritic Cell
Flk2	Fetal Liver Kinase-2
Flt3	Forms Like Tyrosine Kinase-3
GM-CSF	Granulocyte Macrophage-Colony Stimulating Factor
IFN	Interferon
IL	Interleukin
IgE	Immunoglobulin E
mDC	Myeloid Dendritic Cells
MHC	Major Histacompatibility Complex
MIP3-b	Macrophage Inflammatory Protein 3
moDC	Monocyte-Derived Dendritic Cells
nsLTP	Non-Specific Lipid Transfer Protein
pDC	Plasmocytoid Dendritic Cells
Th cells	Helper T Cells
TCR	T Cell Receptor
TLR	Toll-Like Receptor
